# A novel approach for automatic visualization and activation detection of evoked potentials induced by epidural spinal cord stimulation in individuals with spinal cord injury

**DOI:** 10.1371/journal.pone.0185582

**Published:** 2017-10-11

**Authors:** Samineh Mesbah, Claudia A. Angeli, Robert S. Keynton, Ayman El-baz, Susan J. Harkema

**Affiliations:** 1 Department of Electrical and Computer Engineering, University of Louisville, Louisville, KY, United States of America; 2 Department of Bioengineering, University of Louisville, Louisville, KY, United States of America; 3 Frazier Rehab Institute, Kentucky One Health, Louisville, KY, United States of America; 4 Department of Neurological Surgery, University of Louisville, Louisville, KY, United States of America; 5 Kentucky Spinal Cord Injury Research Center, University of Louisville, Louisville, KY, United States of America; University of California Los Angeles, UNITED STATES

## Abstract

Voluntary movements and the standing of spinal cord injured patients have been facilitated using lumbosacral spinal cord epidural stimulation (scES). Identifying the appropriate stimulation parameters (intensity, frequency and anode/cathode assignment) is an arduous task and requires extensive mapping of the spinal cord using evoked potentials. Effective visualization and detection of muscle evoked potentials induced by scES from the recorded electromyography (EMG) signals is critical to identify the optimal configurations and the effects of specific scES parameters on muscle activation. The purpose of this work was to develop a novel approach to automatically detect the occurrence of evoked potentials, quantify the attributes of the signal and visualize the effects across a high number of scES parameters. This new method is designed to automate the current process for performing this task, which has been accomplished manually by data analysts through observation of raw EMG signals, a process that is laborious and time-consuming as well as prone to human errors. The proposed method provides a fast and accurate five-step algorithms framework for activation detection and visualization of the results including: conversion of the EMG signal into its 2-D representation by overlaying the located signal building blocks; de-noising the 2-D image by applying the Generalized Gaussian Markov Random Field technique; detection of the occurrence of evoked potentials using a statistically optimal decision method through the comparison of the probability density functions of each segment to the background noise utilizing log-likelihood ratio; feature extraction of detected motor units such as peak-to-peak amplitude, latency, integrated EMG and Min-max time intervals; and finally visualization of the outputs as Colormap images. In comparing the automatic method vs. manual detection on 700 EMG signals from five individuals, the new approach decreased the processing time from several hours to less than 15 seconds for each set of data, and demonstrated an average accuracy of 98.28% based on the combined false positive and false negative error rates. The sensitivity of this method to the signal-to-noise ratio (SNR) was tested using simulated EMG signals and compared to two existing methods, where the novel technique showed much lower sensitivity to the SNR.

## Introduction

Previously, it has been shown that epidural electrical stimulation in combination with locomotor training and/or pharmacological interventions in animal models were able to highly promote spinal circuits functionality after complete spinal cord transections in rats [1,2]. Subsequently, in the past several years, clinical studies have also reported that lumbosacral spinal cord epidural stimulation (scES) combined with activity-based training progressively re-enabled full weight bearing standing [3,4] and volitional control of lower limbs in individuals with chronic complete paralysis [5,6]. Remarkably, the appropriate selection of stimulation parameters (amplitude, pulse width, frequency and anode/cathode assignment) was shown to be critical to promote the generation of effective motor patterns [3]. Mapping experiments were initially performed with participants in supine position, recording motor evoked potentials from different lower limb muscles using surface electromyography (EMG) during scES with different sets of electrode configurations. The purpose of these experiments was to study the topographical features of recruiting leg muscles by scES [7] and also to provide useful information for the selection of electrode configurations applied for promoting lower limb motor function. The task of determining the links between scES parameters and the characteristics of the evoked potentials is referred to as the “mapping” task in this study.

To study the characteristics of the scES induced evoked potentials, the first step is to localize them inside raw EMG signals that are recorded from several leg muscles by segmenting each EMG signal based on the stimulation onset. One of the most challenging tasks in EMG analysis in the scES content is the precise detection of each epidurally evoked potential. This task is crucial in order to determine the effective threshold for scES intensity that triggered the occurrence of the first visible evoked potential for each muscle. The evoked potential (activation) detection is usually performed manually by a trained observer visually inspecting the raw EMG signals, which is considered to be the most accurate method for activation detection. However, it is a laborious task when facing a large stack of data recorded from several muscles during various experiments. Moreover, manual method can be prone to human errors and inter/intra-observer variation and would also limit the ability to allow scalability to a high number of patients. Therefore, to facilitate the activation detection process, an accurate computer-based method is proposed in this work.

There have been several methods proposed for computer-based change detection for EMG signals in the literature, such as the single or double threshold detector [8], Teager–Kaiser Energy Operation [9–11], wavelet template matching [12], supervised and unsupervised learning algorithms [13] or statistical criterion determination methods like hidden Markov models [14] and Gaussian mixture models [15,16]. The main goal of all these methods is to convert the original raw signals into a set of estimated sequences that make the highest distinction between before and after change as well as detect the occurrence of the change and the corresponding time instant, t_0_, as early as possible [15].

Most of the automatic onset detection methods can be divided into four main stages: pre-processing, conditioning, decision thresholding, and post processing. Most methods have a pre-processing stage for filtering the raw signal with a band-pass filter in order to remove artifacts and reduce the noise level in the signal. In the conditioning stage, the EMG signal passes through a test function, i.e. a type of event indicator. In the third stage, the algorithm will set a threshold that indicates the first point of the signal change. Finally, the last stage deals with the false alarms by setting certain constraints on the detected onset values [15,17]. Most of the methods differ based on the type of test function; the decision rule, which involves the selection of constant or dynamic thresholds; and the heuristic constraints for the final detected onset, which varies based on the application and the characteristics of the EMG signals. There are several categories of event indicator functions, such as on-line vs. off-line, or supervised versus unsupervised learning algorithms. If an algorithm has been executing the task in a sequential fashion for each incoming data point, the method is called on-line; otherwise it is considered off-line [15]. Also, if the training input data for a learning method is already labeled using a priori information, the method is supervised, and if the algorithm estimates a model for the input data using specific parameter estimation techniques, it is an unsupervised technique.

In every activation detection method, sensitivity to the noise level in the signal is a great challenge. Selection of proper pre- and post-processing methods usually helps to minimize the effect of noise on the accuracy of the method. It is also notable that some of the proposed methods are highly dependent on prior information of the signal, i.e. supervised methods, which makes those methods semi-automatic and their accuracy application-dependent. In this study, we suggest a novel method to address these two main challenges that a fully automatic activation detection method faces, by proposing an unsupervised and on-line approach that deals with the stochastic characteristics of the EMG signals in the scES application.

The main purpose of this work is to develop a novel method for automatic detection of the epidurally evoked potentials using a generalized framework to perform the scES-EMG mapping task. The generalized framework will: 1) effectively de-noise, detect, and extract the key features of the signal; 2) visualize the occurrence of the muscle evoked potentials induced by scES; and, 3) increase the accuracy and efficiency of the physiological mapping process in order to determine the underlying relationships between the scES parameters and muscle activations. Consequently, this framework will assist the data analysts to promptly decide on further adjustments or improvements in designing future experiments.

## Materials and methods

### Participants

In this study, five male individuals with motor complete spinal cord injury (SCI) have participated. Two of these participants have American Spinal Injury Association Impairment Scale (AIS) grade B and three of them have AIS grade A. The average age of these five individuals at the time of the experiments was 29.8 ± 4.5 years old and the average time since injury was 4.2 ± 1.6 years. All five participants have provided written, informed consent for the experimental procedures, which have been approved by the University of Louisville Institutional Review Board.

### Spinal cord epidural stimulation

A stimulation unit (RestoreAdvanced Neurostimulator, Medtronic, Inc., Minneapolis, MN) in combination with a chronic, 16-electrode array (39565 paddle electrode array, Medtronics, Inc., Minneapolis, MN) is surgically implanted at the T11–L1 vertebral levels over the spinal-cord segments L1–S2. It is used to deliver electrical stimulation to the lumbosacral spinal cord of each SCI individual. The electrode array was connected to the IPG unit that was implanted in a subcutaneous abdominal pouch [5].

### EMG recording system

**Fig 1:**
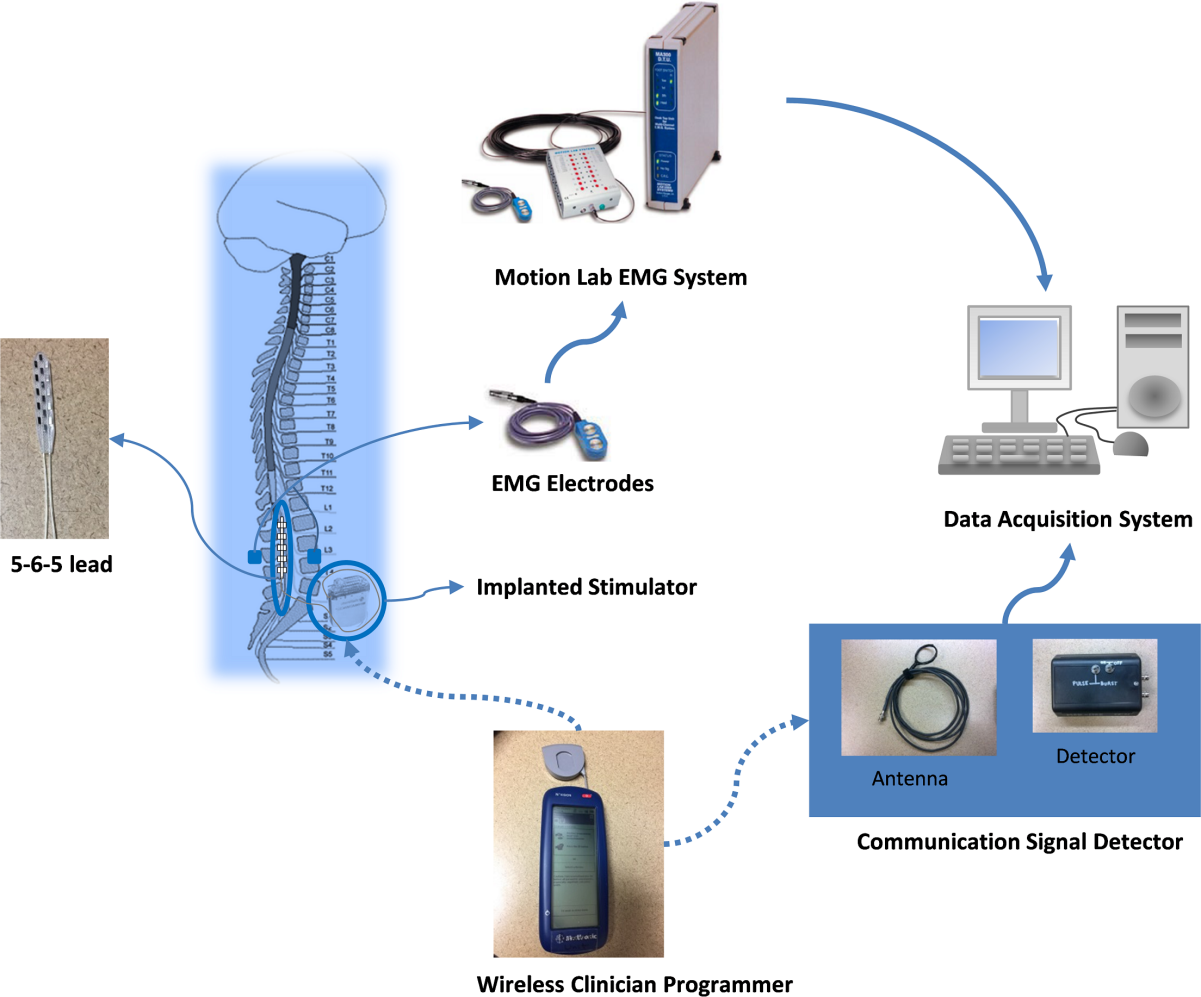
Schematic representation of the epidural stimulation unit (16-electrode array, IPG unit and wireless programmer) and its connections to the EMG recording system. (The Motion Lab EMG System and EMG electrodes illustrations are from Motion Lab System Inc. Manual).

National Instrument Data Acquisition system (National Instruments, Austin, TX) was built to collect both EMG signal and the signal from the communication signal detector. EMG signals were recorded and filtered (band-pass filter of 10 Hz–2 kHz (−3 dB)) with differential surface electrodes (Motion Lab Systems, Baton Rouge, LA) from Motion Lab MA300 EMG system. Two surface electrodes were placed symmetrically lateral to the electrode array incision site over the paraspinal (PS) muscles in order to record the stimulation artifacts. Other electrodes are placed to record 14 thigh and leg muscles signal. A communication signal detector was developed to capture the communications signal (stimulation parameters) between the Clinician Programmer and IPG. The communication signal detector sends detected stimulation parameter change to the data acquisition system. Using the PS EMG artifact signal and the captured communication signal to mark the onset of each stimulation pulse [7]. Fig 1 illustrates the connections between the epidural stimulation unit and the EMG recording system.

### EMG data acquisition

The scES lower limbs mapping experiments start 2–3 weeks after the surgical implantation [5,7]. The supine experiments are performed in accordance with the procedures previously described by [5] with the participants relaxed in a supine position. The electrical stimulation waveforms are comprised of a rectangular, biphasic shape with pulse duration of 450 μs. In the supine experiments, specific combinations of electrodes are selected for activation, which is referred to as stimulation configuration. A total of 12 different stimulation configurations are examined for each individual. For each configuration, stimulation intensity or frequency will be increased whilst the other parameters are fixed. The stimulation voltage (intensity) ramp-up experiments are performed with the scES intensity (in volts) gradually increased and the frequency is set at 2 Hz. During each intensity ramp-up, the scES intensity starts at a pre-activation value (V) and increases at either 0.1 or 0.5 V increments up to 10 V with time interval between each ramp-up at least 2–3 seconds, which in this time interval scES delivers a minimum of five stimulus pulses for each intensity level (at 2 Hz). In the frequency ramp-up experiments on the other hands, after the intensity is set at the value where all the muscles are activated (*f* = 2Hz), the frequency is increased from 2 to 5 Hz and from 5 to 60 Hz with the step 5 Hz.

During the performance of each experiment, the surface EMG signals are recorded from 14 leg muscles, using bipolar surface electrodes that are placed on the left (L) and right (R) soleus (SOL), medial gastrocnemius (MG), tibialis anterior (TA), vastus lateralis (VL), rectus femoris (RF), medial hamstrings (MH), and gluteus maximus (GL). The recorded EMG signals are digitized at a sampling rate of 2000 samples per second. The heart rate and blood pressure data of each participant are also recorded during the experiments.

### Methodology

**Fig 2:**
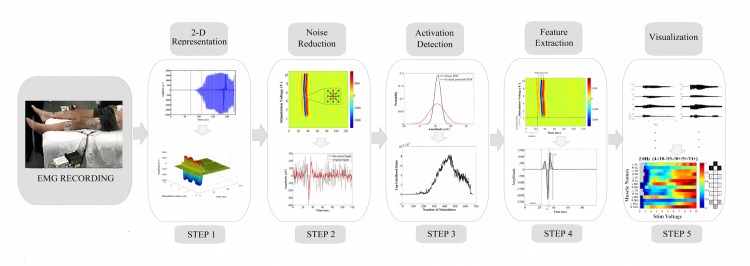
Block diagram of the proposed framework for visualization and activation detection of evoked potentials induced by scES.

In this study, a set of five algorithms is proposed to perform the mapping task in an automated fashion to convert the raw recorded EMG signal into its significant building blocks, i.e. the evoked potentials induced by scES. Additionally, the algorithms extract several key features of these evoked potentials, such as peak-to-peak amplitude, latency, integrated EMG and Min-max time intervals, and enable visualization of these features to effectively represent the desired hidden information in the EMG recordings to the data analysts. Fig 2 illustrates the block diagram of the general framework, and the pseudocode for each step of the framework is presented in the supplementary materials, S2 Appendix.

#### 2-D representation of EMG signal

**Fig 3:**
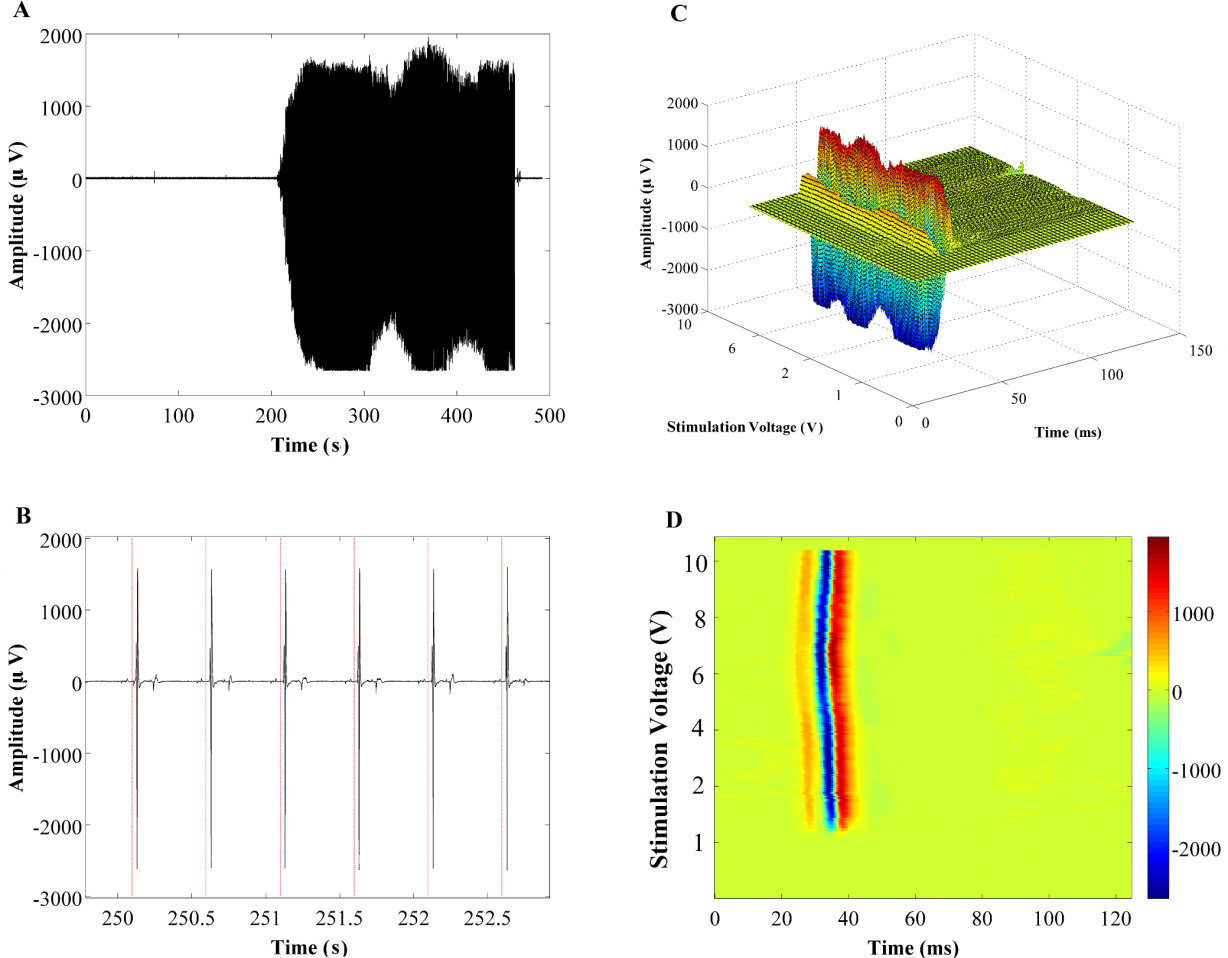
The steps for converting raw EMG signals into 2-D and 3-D images. (A) Raw EMG signal, (B) Signal segmentation using stimulation time intervals, (C) Overlaying all the segments and building the 3-D graph where X-axis is the evoked potentials duration (ms), the Y-axis is the stimulation voltage (V), and the Z-axis is the amplitude of the signals (μV) and (D) Converting the 3-D graphs into 2-D images using Colormap.

From a computational point of view, each single record of the EMG (*x*_*k*_,*k* ≥ 1) is a set of sample observations from a discrete random process (*X*_*k*_,*k* ≥ 1). The EMG signals, which are recorded from all 14 leg muscles, consist of evoked potentials (*w*_1_,*w*_2_,…,*w*_*N*_ ∈ *W*) that are induced by ES. Utilizing the onsets of the stimulation pulses, the whole EMG signal is segmented into its building blocks (*W* set) where each segment consists of the time interval between two consecutive stimulation pulsations (Fig 3A and 3B). Subsequently, the first algorithm converts the EMG signal, *X*_*k*_, to a 3-D image, *δ*_*k*_(*x*,*y*,*z*), by overlaying all the EMG signal segments and then displaying their value in 3-D graphs (Fig 3C). These 3-D graphs are converted to 2-D images using Colormap algorithm where the amplitude values are represented as colors (Fig 3D). Each row in the Colormap image illustrates one segment of the whole EMG signal (Algorithm I in S2 Appendix). Fig 2 demonstrates all the steps for this conversion process for a sample EMG signal.

#### Noise reduction

After signal to image conversion, it is possible to use image-processing techniques for smoothing the images and consequently de-noising the signals. In this study, a 2-D generalized Gaussian Markov Random Field (GGMRF) model is applied to the constructed 2-D images so as to reduce the noise level from the EMG signal [18]. This particular smoothing method preserves continuity and removes inhomogeneity in the image, which in this context is caused by background noise in the original EMG signals. This is achieved by comparing each pixel’s value, which is the evoked potential amplitude in μV, to the n-neighborhood pixel set and recalculating the respective pixel value based on Eq 1.

$${\hat{\delta }}_{s}={\mathrm{argmin}}_{\delta_{s}}\left\{{\left|\delta_{s}-{\tilde{\delta }}_{s}\right|}^{q}+\sigma^{q}\lambda^{p}\sum_{r\in \upsilon_{s}}b_{s,r}{\left|{\tilde{\delta }}_{s}-\delta_{r}\right|}^{p}\right\}$$ (1)

**Fig 4:**
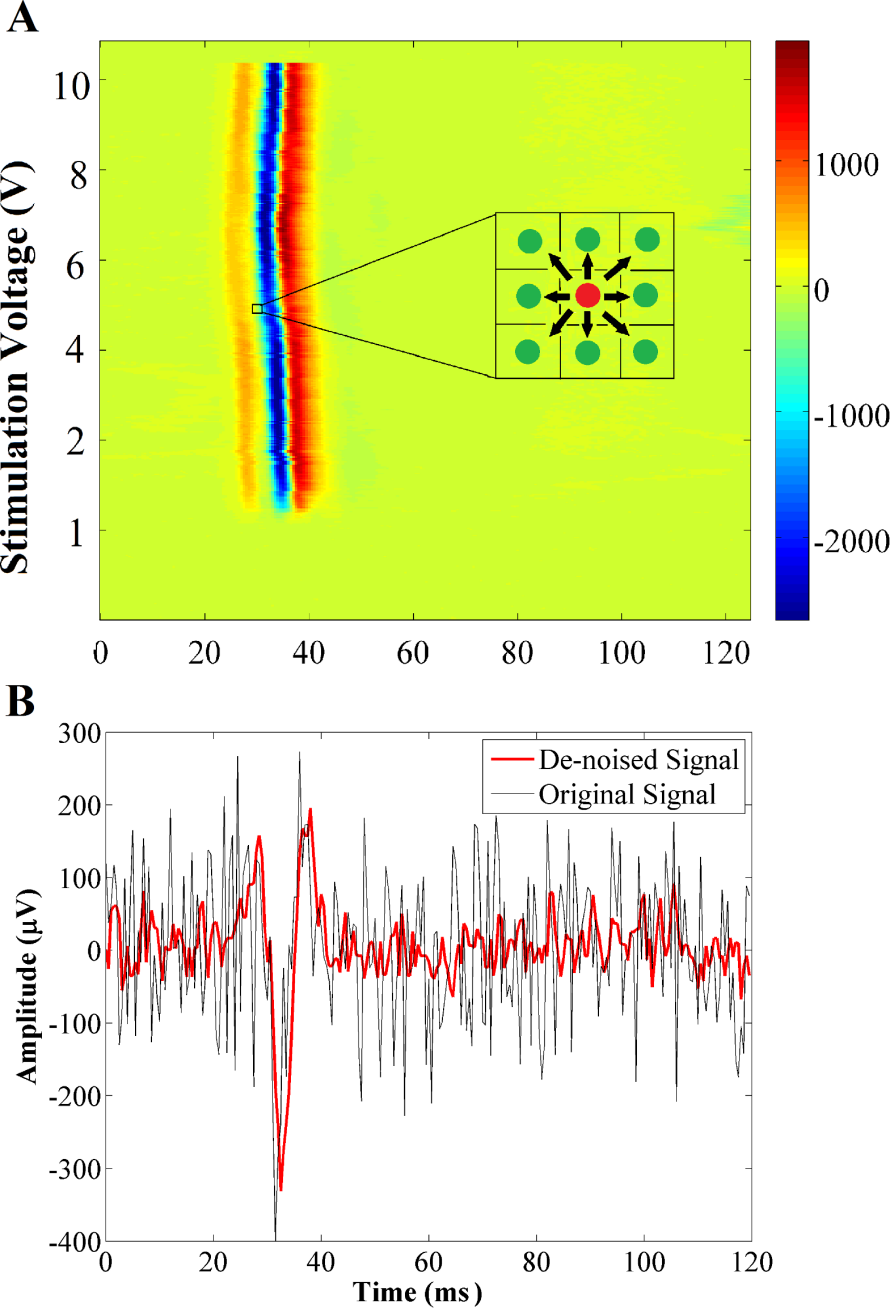
EMG denoising using GGMRF. (A) Applying GGMRF method to 2D image (B) An example of evoked potential before (black) and after (red) applying GGMRF method.

Where ***δ***_***s***_, ${\hat{\delta }}_{s}$ and ${\tilde{\delta }}_{s}$ are the original pixel value, its recalculated value, and expected estimate, respectively. ***υ***_***s***_ is the 8-neighborhood pixel set; ***b***_***s*,*r***_ is the GGMRF potential; and ***σ*** and ***λ*** are scaling factors. The parameter ***p*** ∈ [1.01, 2.0] controls the smoothing level (e.g., ***p*** = 2 for smooth versus ***p*** = 1.01 for relatively abrupt edges). The parameter ***q*** ∈{1, 2} determines the Gaussian (***q*** = 2) or Laplacian (*q* = 1) prior distribution of the estimator [19]. Our simulations are conducted with ***σ*** = 1, ***λ*** = 5, ***p*** = 1.01, ***q*** = 2, and $b_{s,r}=\sqrt{2}$ (see [18] for more details on GGMRF). The size of the neighborhood, n, has a great impact on the level of smoothing and needs to be adjusted for each application. An example of the input and output of the GGMRF method and their corresponding muscle activation segments is demonstrated in Fig 4 (Algorithm II in S2 Appendix).

Advantages of spatial smoothing of the EMG signals compared to the traditional band-pass filtering techniques, is that this method reduces EMG signal variability by offering the option of comparing each evoked potential with the previous and next one. Unlike the filtering methods, this image smoothing method offers a de-noised signal without any significant changes in the position or original shape of muscle activations.

#### Activation detection

The activation detection algorithm is designed to determine the presence or absence of scES induced evoked potentials in each segment of the EMG signals and, consequently, the corresponding stimulation intensity threshold. The pre-assumptions for this task are: 1) the intensity threshold V_s0,_ which causes the emergence of the earliest evoked potentials, is an unknown random value with unknown distribution; and, 2) the amplitude of the first visible evoked potential is also an unknown value. These are valid assumptions because of the non-stationary nature of EMG signals and the fact that no *a priori* information regarding the distribution of the varying parameters is present. It is notable that these onset values alter based on the choice of the stimulation configuration, frequency and muscle type, and also subject to the day-to-day and pre-post training variability. As mentioned in section 2.1, scES delivers the minimum of five stimulation pulses per stimulus voltage referred to as an *event*, (*w*_*j*_, *L*_*n*−1_ ≤ *j* ≤ *L*_*n*_) where min(*L*_*n*_ − *L*_*n*−1_) = 5. It is defined by clinical analysts that if 50 percent or more of the stimulation pulses corresponding to the same intensity (event) trigger evoked potentials, that intensity will be considered as the activation threshold voltage (V_s0_).

**Fig 5:**
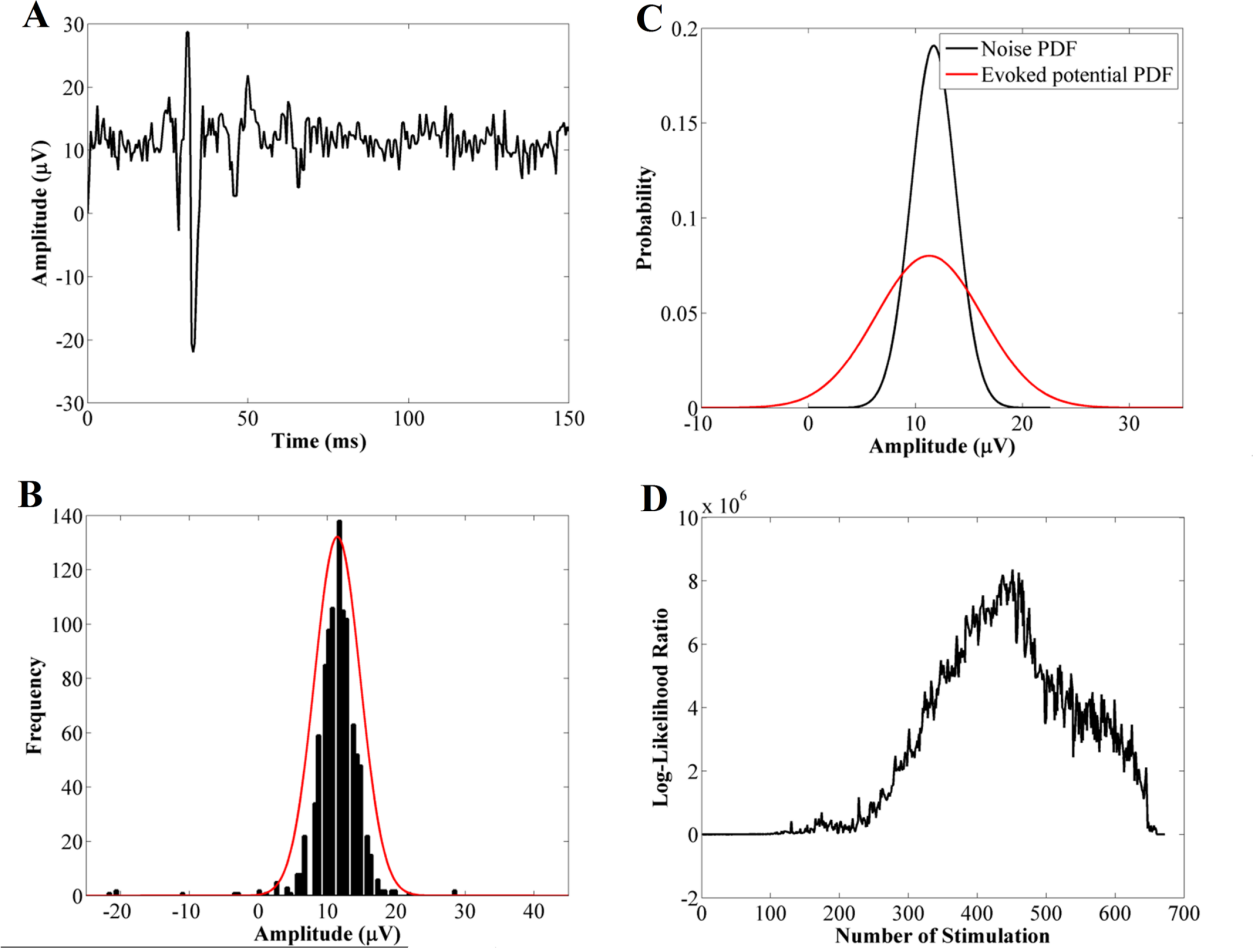
Calculation steps for activation detection algorithm. (A) Sample evoked potential (one segment of the EMG signal), (B) Histogram of the sample evoked potential (black) and its estimated Gaussian distribution (red), (C) Comparing the Gaussian pdf of evoked potential signal (red) to pdf of background noise (black), (D) Plotting the calculated LLR for all segments of the EMG signal and detect the activation threshold (arrow).

The general technique implemented in this step is known as the statistically optimal decision (SOD) method. One of the well-known derivations of SOD is named approximated generalized likelihood-ratio (AGLR) detector that is developed by Staude and Wolf (1999). In this study, this method is modified and adapted to our activation detection application. There are three main phases to this activation detection method: in the first phase, each segment of the de-noised signal (*w*_*i*_ ∈ *W*) is modeled by a Gaussian probability density function (pdf) and the model parameters *μ*_*i*_ and *σ*_*i*_ are estimated using the maximum likelihood estimation (MLE) method (*p*_*θ*_(*w*_*i*_)). Fig 5B shows the histograms for one segment of an EMG and its estimated Gaussian distribution. From a statistical point of view, the activation detection method represents a binary selection between the null hypothesis H_0_ that states “there is no significant change in the pdf $p_{\theta_{0}}(w_{i})$ of the *i*^*th*^ segment of the signal” and the alternate hypothesis that states “there is a significant change in the parameters of pdf $p_{\theta_{1}}(w_{i})$” [15]. Therefore, in the second phase, the Gaussian model of all the EMG segments will be compared to the Gaussian model of the background noise by the log-likelihood ratio (LLR) measure. Eq 2 shows the general formulation of the LLR.

$$s_{k}=\ln\left(\frac{p_{\theta_{1}}(y_{i_{k}})}{p_{\theta_{0}}(y_{i_{k}})}\right)$$ (2)

Where $y_{i_{k}}$ is the *k*^*th*^ sampled-value of *w*_*i*_ segment after smoothing step. In order to reduce the high computational cost of this equation, it is assumed that the occurrence of the scES induced muscle activation does not change the mean *μ* of the Gaussian pdf and the most significant changes happen in the standard deviation *σ*_*i*_ of the pdf as shown in Fig 5C. Therefore, the Eq 2 can be simplified to Eq 3.

$$s_{k}=\ln(\frac{\sigma_{0}}{\sigma_{i}})+\frac{1}{2}{\left(y_{i_{k}}-\mu \right)}^{2}(\frac{1}{{\sigma_{0}}^{2}}-\frac{1}{{\sigma_{i}}^{2}})$$ (3)

The sum of all the *s*_*k*_ values over one segment is referred to as CUSUM value *S*_*i*_ and is calculated based on Eq 4.

$$\begin{array}{l} S_{i}=\sum_{k=N_{i-1}}^{N_{i}}s_{k}\\ =(N_{i}-N_{i-1}+1)ln\left(\frac{\sigma_{0}}{\sigma_{i}}\right)+\frac{\left(N_{i}-N_{i-1}\right)}{2}(\frac{{\sigma_{i}}^{2}}{{\sigma_{0}}^{2}}-1) \end{array}$$ (4)

Using Eq 4, for each segment of the signal one value is calculated, which represents the highest statistical difference between that specific segment and the background noise (Fig 5D).

Finally, in the third phase of the algorithm, a dynamic threshold *h* is calculated for each EMG signal in order to find the first segment of the signal that includes the evoked potential and its corresponding stimulation intensity V_s0_. Based on experimental observations, the first event corresponding to the lowest stimulation voltage does not usually trigger muscle activation; thus, this event can be considered as the baseline. Consequently, the threshold value *h* can be computed as the summation of the maximum and standard deviation of the set of *S*_*i*_ that belongs to the baseline (Eq 5).

$$h=S_{max}+\sigma_{S_{i}}$$ (5)

All the steps of the activation detection method are demonstrated in Fig 5 (Algorithm III in S2 Appendix). This step of the framework has been applied only to the voltage ramp-up experiments (at 2 Hz) where there is a single evoked potential in response to each stimulation pulse (after the muscle is activated) and the accurate detection of the muscle activation and its onset is desired.

#### Feature extraction

**Fig 6:**
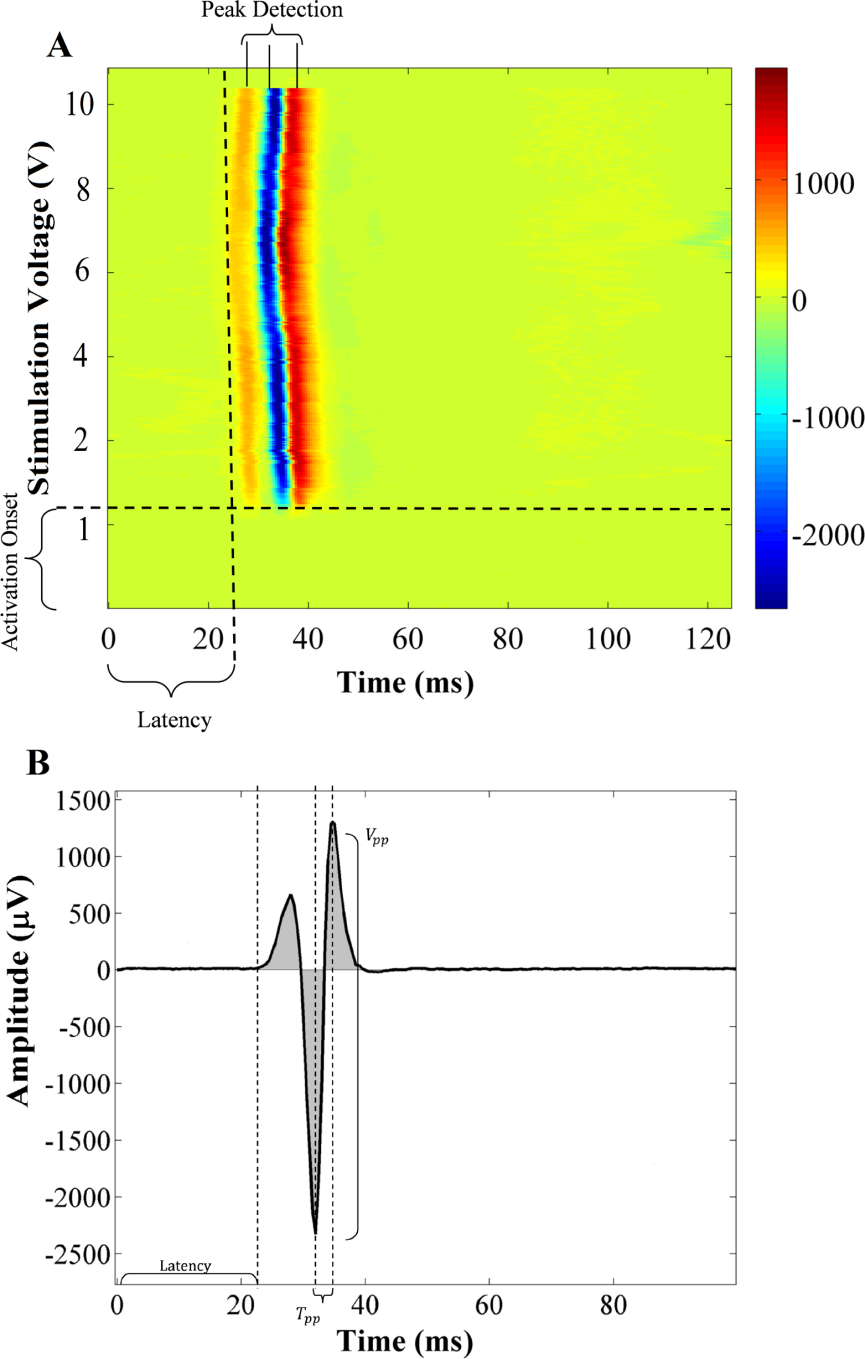
Selected feature parameters for EMG activation signal. (A) Visual inspection: Number of peaks of the evoked potential, Activation onset and Latency, (B) Computer-based feature extraction of peak-to-peak amplitude (V_pp_), Activation latency, Time interval between minimum and maximum values (T_pp_) and Integrated EMG (summation of absolute values of all gray areas).

The objective of the feature extraction algorithm is to represent each epidurally evoked potential with a set of key features. With this algorithm, the user has the flexibility to calculate these parameters automatically or observe visually using the 2-D representation of the EMG signal (Fig 6A). The automatically calculated parameters in this framework are: peak-to-peak amplitude (V_pp_), which is the absolute value of the difference between the highest and the lowest peaks (T_pp_) in the evoked potentials and its normalized value based on the highest peak between left and right muscle; activation latency, the time interval between the stimulus onset and the onset of muscle activation, the time interval between the highest and lowest peak; integrated EMG value, the area under the motor unit curve after rectifying the EMG signal; and, finally, binary 0/1 values: an indication of the absence or presence of evoked potentials in each segment of the signal (Algorithm IV in S2 Appendix). The aforementioned features are illustrated in Fig 6B.

#### Visualization

**Fig 7:**
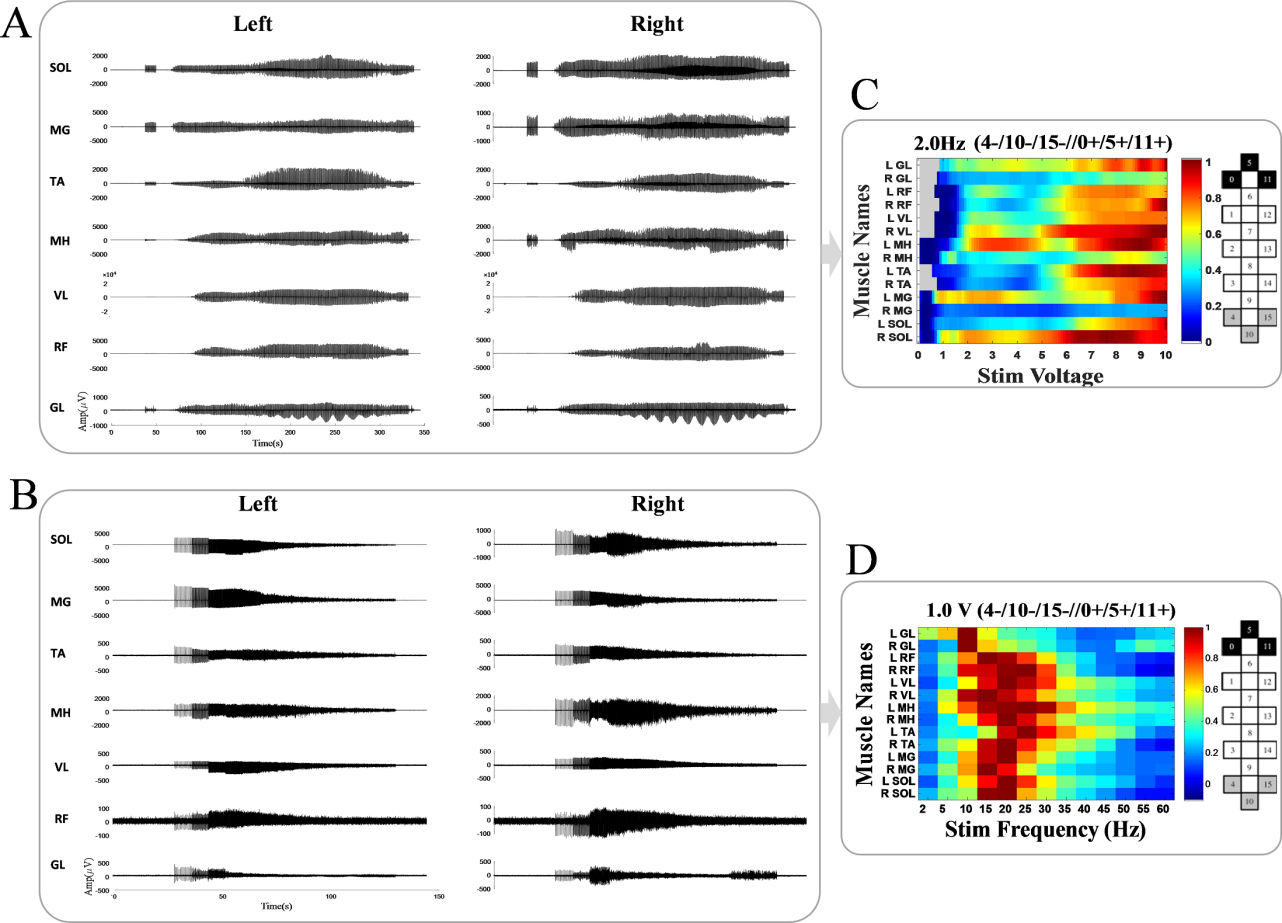
Examples of converting 14 EMG signals into colormap images for voltage ramp-up and frequency ramp-up experiments. (A) Raw EMG signals of 14 ploximal and distal left and right leg muscles during voltage ramp-up from 0.1 to 10 V. (B) Raw EMG signals of same muscles during frequency ramp-up from 2 to 60 Hz. (C) Colormap image shows the corresponding peak-to-peak amplitudes (*μV*) with respect to each muscle and stimulation voltage after stimulation threshold detection. The gray area is presenting the pre-threshold part of the experiment where no activation was induced. (D) Colormap image shows the corresponding integrated EMG values with respect to 14 muscles and stimulation frequencies.

The last step of the framework is to represent the data processing results in an optimum and informative way to illustrate the connection between stimulation parameters and results generated from the computer-based method for each muscle. This will create a valuable, efficient and convenient presentation of the data for the examiner to enhance interpretation and modify the experiments accordingly. Fig 7 shows examples of the transformation of 14 raw EMG signals into a single Colormap image for intensity ramp-up (Fig 7A and 7C) and frequency ramp-up experiments with the same stimulation configuration (Fig 7B and 7D). The Colormap values represent the peak-to-peak amplitudes for intensity ramp-up, after the automatic detection of scES-induced activation, and integrated EMG values for frequency ramp-up experiments.

The colormap visualization technique can be expanded for several ramp-up experiments where different configurations are tested for each subject. This is particularly helpful for the experimental analysts since it gives them the option for instantly comparing the results of several experiments together and deside on the optimum stimulation parameters selection. Examples of this form of colormap representation are demonstrated in the results section.

## Results

In this section, the accuracy and speed of automating the mapping task is presented followed by a few examples of the clinical applications of the proposed framework. The performance of the computer-based activation detection algorithm has been evaluated by comparing the output of the algorithm with the output of the manually detected evoked potentials in intensity ramp-up experiments performed by trained data analysts, which is considered as the gold standard. The activation detection method presented is also compared with two other existing methods, Teager-Kaiser Energy Operation (TKEO) [9] and the AGLR method without the GGMRF smoothing step. The comparison is based on both recorded EMG signals and simulated signals. Finally, the runtimes of all the algorithms are presented.

### Performance evaluation using manual activation detection

**Fig 8:**
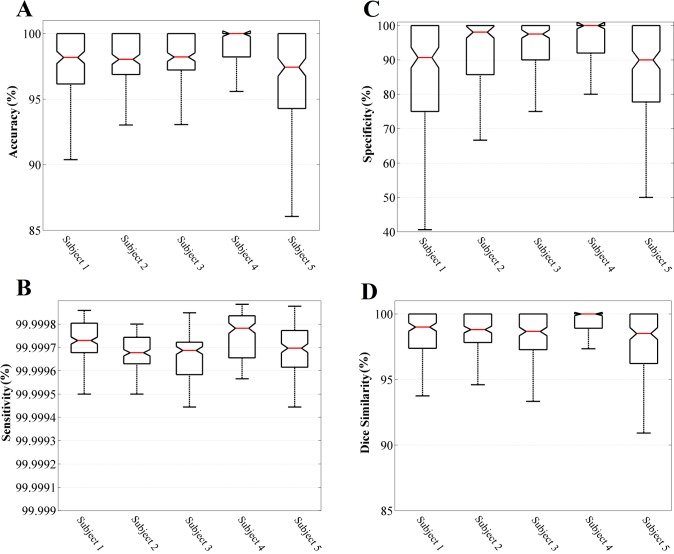
Boxplot representation of performace measurements for comparing automated activation detection method with the ground truth. (A) Accuracy, (B) Sensitivity, (C) Specificity, (D) Dice Similarity.

This evaluation is based on calculating sensitivity, specificity, Dice similarity and accuracy as the performance metrics. These parameters are calculated based on true positive (TP), true negative (TN), false positive (FP), and false negative (FN) values. The boxplots of the four performance measurements on 700 EMG signals separated for each individual are shown in Fig 8.

The mathematical equations for calculating these parameters and overall values from comparing automatic vs. manual activation detection method are presented in S1 Table.

Looking at these values in S1 Table and Fig 8, it is immediately noticeable that the selected comparison measures (i.e. sensitivity, specificity, Dice similarity and accuracy) have distributed in different ranges. For instance, the percentage of sensitivity values are densely distributed in [99.9994, 99.9999] interval whereas the specificity data points are distributed in [79.59, 99.99] or accuracy values are spread out between [94.33, 100.00]. These differences can be addressed based on the experimental design and the original definitions of these measures. For example, the sensitivity is the ratio of true positives over all the positive (activated) segments of the signal, and since during the intensity ramp-up usually most segments of the EMG signals contain evoked potentials, the true positive ratio is almost always close to 100%. On the other hand, the specificity values show the ratio of true negatives and since a few segments usually fall into the not-activated category, the accuracy of the program for detecting these segments can drop in some cases and cause in lower true negative ratio. The Dice similarity measure is usually used to quantify the amount of agreements between two sets of binary results and in this application these values are distributed in [96.29, 99.99].

The five-number-summary values of the performance measures classified by *subjects* and *muscles* presented in supplementary materials, S1, S2 and S3 Tables, respectively.

### Comparison with other activation detection methods

The performance of the automated method is compared to two other methods: TKEO and AGLR without smoothing. In this study, we slightly modified these algorithms to be adapted to the activation detection problem in order to make a fair comparison between their outputs and our proposed method. The TKEO method utilizes a conditioning function as shown in Eq 6.

$$\Psi (x_{i_{k}})={{x_{i}}_{k}}^{2}-{x_{i}}_{k-1}{x_{i}}_{k+1}$$ (6)

Where ${x_{i}}_{k}$ is the observation *k* in segment *i*. After applying the conditioning function, the maximum value of each segment is compared to the dynamic threshold (Eq 5) and activation will be detected if the maximum value is greater than the threshold.

The comparison results are based on recorded EMG signals as well as simulated EMG signals. The pseudocode of the TKEO method implementation is presented in the supplementary materials, S2 Appendix, Algorithm VI.

The results of comparing the proposed method with AGLR and TKEO based on the total accuracy in the recorded EMG signals from five patients are presented in Table 1 in the five-number-summary format. It is noticeable that AGLR and TKEO showed a lower accuracy compared to the new automated framework. Particularly there is approximately 1.05% increase in the median value of accuracy that shows the effect of adding the GGMRF smoothing technique to the pre-processing step, which makes the automated method more robust where there is higher noise level in the signal. It is notable here that although other signal filtering techniques might show the same robustness to noise, these methods cause distortion to the shape of the evoked potential or activation latency in the signal which are unfavorable in this application.

**Table 1:** Comparison of the total accuracy for the new automated activation detection method with the TKEO and SODM methods based on five-number-summary.

	SODM+GGMRF	SODM	TKEO
**Maximum**	100.00	100.00	100.00
**Upper quartile**	100.00	100.00	100.00
**Median**	100.00	98.95	98.42
**Lower quartile**	97.72	97.43	97.22
**Minimum**	94.33	93.65	93.18

**Fig 9:**
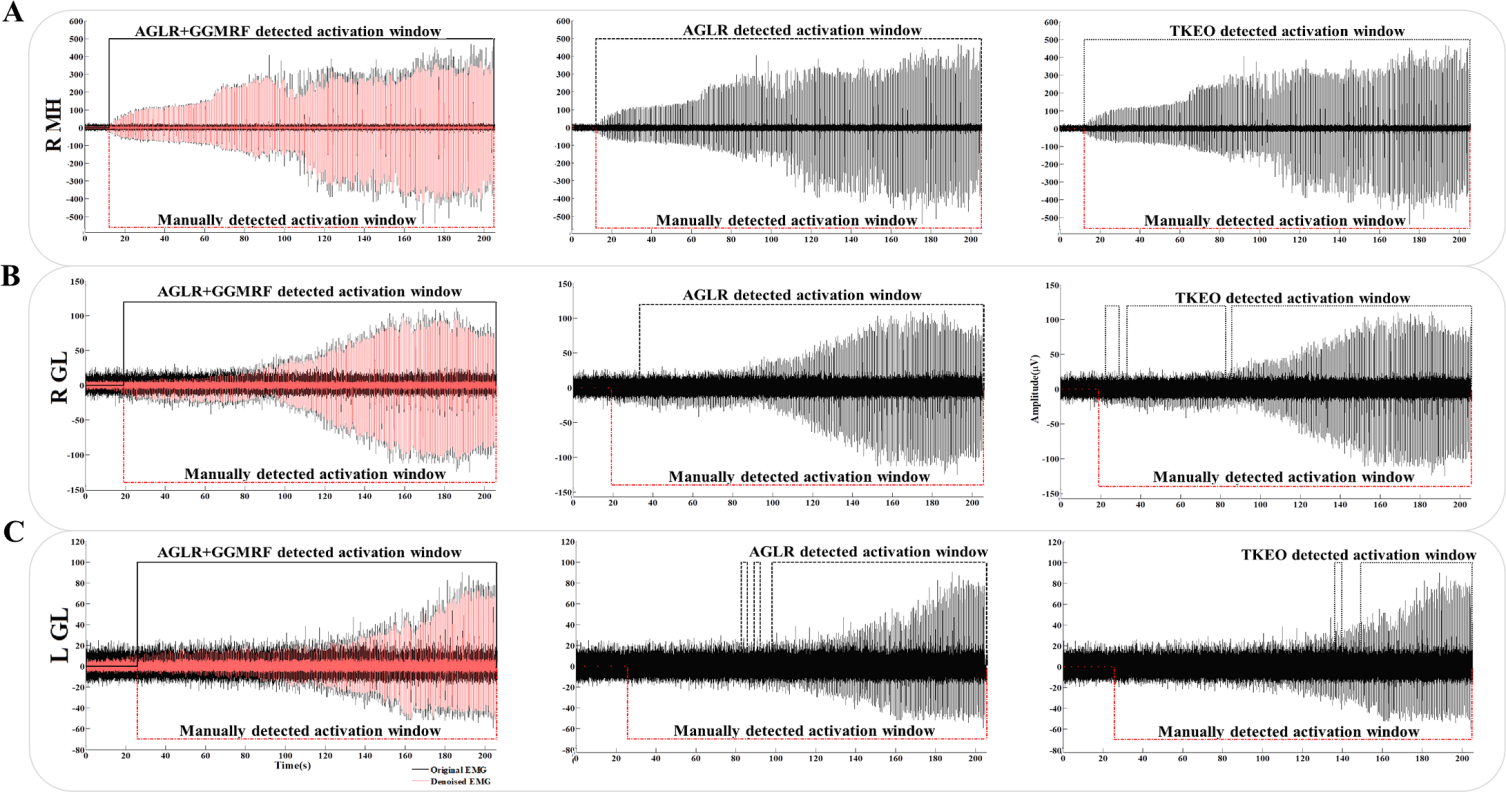
Examples of recorded EMG signals with different SNR levels and the performance comparison between three activation detection methods. (A) High SNR signal from right MH; (B) Medium SNR signal from right GL and (C) Low SNR signal from L GL. Detected activation windows for AGLR + MMGRF, AGLR and TKEO from left to right are shown as continues and dashed black lines. De-noised signal is shown in light red and manually detected activation window as dashed red line.

Fig 9 demonstrates examples of recorded EMG signals with different SNR levels and performance comparison of the three methods for each signal. The three signals were recorded from right MH (Fig 9A), right GL (Fig 9B) and left GL (Fig 9C) during an intensity ramp-up experiment and they have high, medium and low SNR (10.63 dB, 4.37 dB and -0.14 dB), respectively. These SNR values are approximated by using the smoothed signals as the original signal without noise and the subtraction of recorded and smoothed signal as the noise signal (assuming that the noise is additive). The performance of the three methods are shown as activation windows in black lines (from left to right: AGLR with GGMRF, AGLR and TKEO). The activation window is a time interval in which the algorithm was able to detect every single evoked potential induced by scES pulsations. The manually detected activation windows are shown in red dashed lines as the ground truth.

As this figure shows, for the high SNR signal all three methods can detect 100% of the evoked potentials in the signal. In the medium SNR, only AGLR with GGMRF can detect the activations as early as the ground truth. The AGLR without de-noising had near 15 seconds delay in detecting the first activation (accuracy of 94.11%). TKEO didn’t show a long delay but it was unable to consistently detect all the activations throughout the signal (accuracy of 95.58%). In the last row, the left GL signal has low SNR and it causes a long delays and inconsistencies in detecting the activations for AGLR (69.11%) and TKEO (38.23%), however our algorithm is robust in detecting the earliest activations that are overwhelmed with the high noise level.

**Fig 10:**
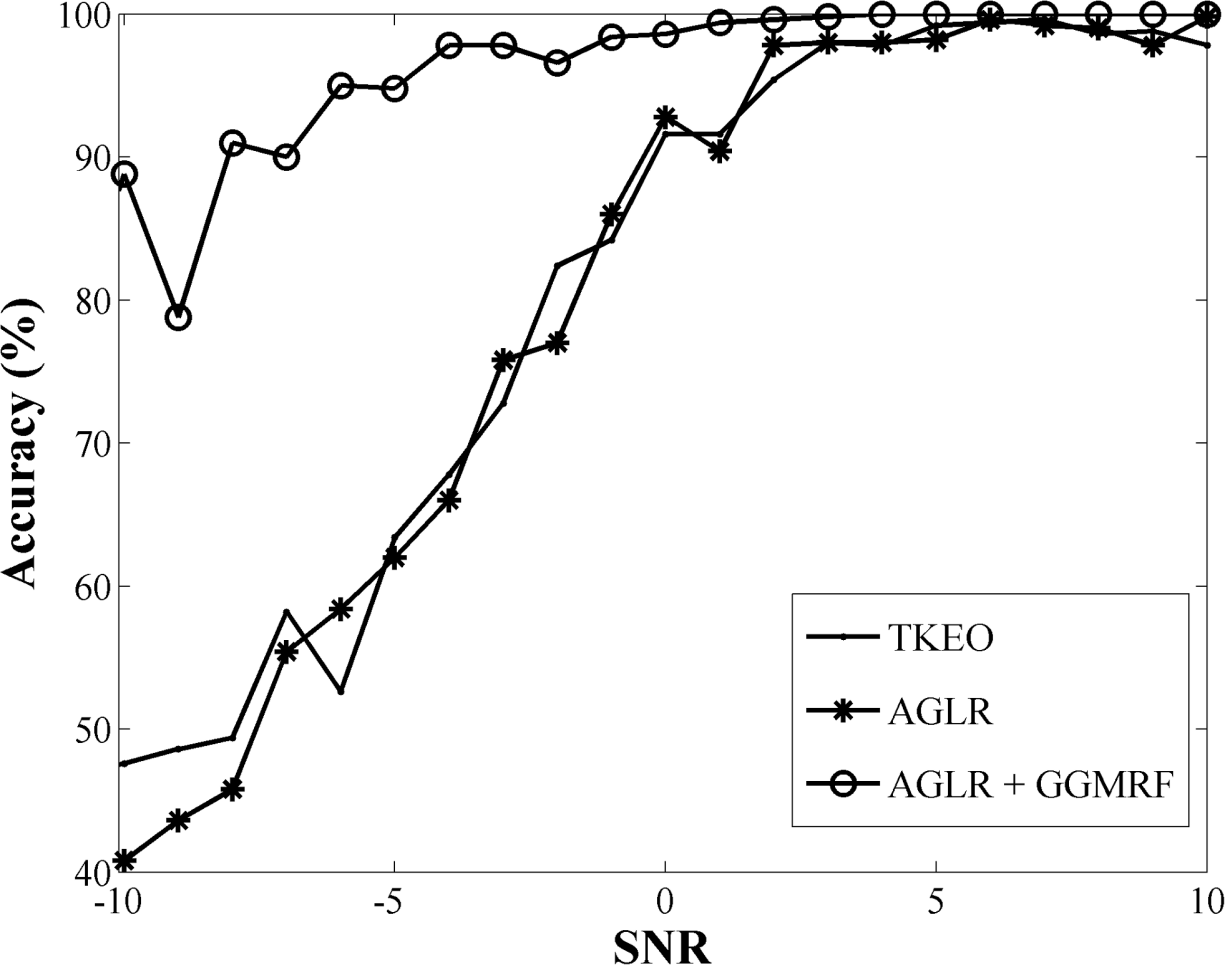
Comparison of three activation detection methods on simulated EMG signals as a function of SNR(dB).

In order to measure to what extent the proposed method keeps its robustness in the noisy environment, and compare it with other methods, the performance of the three methods were tested using the simulated signals with different SNR values. The simulated EMG signal is designed using an activation shape signal as the evoked potential, X(k), that is convolved with a train of Dirac delta impulses $\sum_{n=1}^{k}0.01n\;\delta (t-n)$, where the amplitudes linearly increase. The additive white Gaussian noise, n(t), is then added to the signal based on the desired SNR to generate the final simulated signal. This signal consists of 50 segments, where the first 20 segments do not include any evoked potential; thus, 10 segments of the first portion of the signal are utilized as the baseline. The SNR of the simulated signal is increased from -10 to 10 dB (S1 Fig). Fig 10 demonstrates the plot of the accuracy measurement values versus SNR for three methods. As it is shown in this figure, all three methods are performing well at higher SNR values. However, as the noise level in the signal started to increase, TKEO and AGLR accuracy rates suddenly dropped to below 50% but the proposed method kept its accuracy near 80% at lower SNRs. Therefore, it is clear from this plot that the AGLR with GGMRF significantly outperforms the other methods for EMG signals containing lower SNR values.

### Calculating total runtime of the algorithms

The hardware and software that were employed to process the EMG signals and calculate the runtime of the algorithms are Dell computer with Optiplex 9020, Intel® Core™i7-4790 CPU @ 3.60 GHz, 16.0 GB RAM, 64-bit Operating System, and MATLAB R2011a, respectively. The runtime of this framework is directly dependent on the length of the experiments. Table 2 presents the average runtime of each of the five algorithms. The average and standard deviation of total execution time for processing each set of data recorded during one ramp-up experiment through all five algorithms is approximately 12.7 ± 2.3 seconds. Comparing to the previous manual method that could take up to several days, the automated method clearly demonstrates superior efficiency with respect to time and human resources.

**Table 2:** The runtime mean and RMSE of each steps of the proposed framework.

		Converting signal to image	Noise reduction	Activation detection	Feature extraction	Visualization	Total
**Runtime (s)**	**Mean**	3.93	6.61	1.54	0.05	0.40	12.71
	**RMSE**	1.33	1.18	0.24	0.01	0.01	2.33

## Discussions

There are several points that need to be addressed in the process of developing the proposed automated framework for EMG signal processing. First, it is important to note that the presented method does not need any *a priori* information for the statistical model, which makes it a fully automatic method that uses only the current and previous EMG signal values to build the statistical model. Also, there is no need to label the input data manually before running the program.

Comparing the results from recorded EMG signals and simulated signals with controllable SNR shows that in most cases surface EMG signals have fairly high SNR, which makes the performance of all the methods fairly accurate. However, there are some instances where the EMG signals can be disturbed with unpredictable sources of noise and artifacts like displacement of the recording electrodes or random occurrence of internal muscle activations that can interfere with the epidurally induced activations. As it was shown in the results section, adding the image-smoothing step to the framework helps to reduce the chance of false alarms (outliers) in the presence of these sources of noise. Since both TKEO and AGLR show rather accurate results without GGMRF, we predict that adding this pre-processing step to TKEO and probably other methods will also increase their accuracy significantly. It should also be mentioned that the GGMRF method itself is sensitive to the size of the neighborhood pixels and this parameter should be adjusted based on the application; therefore. adding this step can be a tradeoff between accuracy and the need for parameter adjustment.

In addition to internal activations there are some instances where there is a secondary (late) response to the scES stimulation that can overgrow the primary evoked potential and cause false alarms. In order to minimize the effects of these secondary responses on the program’s accuracy, only the first one eighth of each signal segments are used to build the statistical model because this is the time interval where primary activations, that are directly linked to the order of muscle recruitment of the scES, are most likely to happen.

As it was explained in the methodology section the program is designed to detect both the occurrence of evoked potentials and the latency (onset) of each detected evoked potential as one of the key features that has been extracted. Same statistical methodology with minor modifications has been applied for the onset detection algorithm as explained in the pseudocode, S2 Appendix. In order to increase the resolution of the detected onsets, the program increases the sampling rate to 10,000 samples per second in the recorded EMG signals using linear interpolation technique.

It should also be noted that while the manual activation detection has been considered as the gold standard in this study, the manual method itself has weaknesses such as disagreements between different observers in low SNRs or intra-observer variability in detecting the true onset that can be caused by exhaustion specially when facing large stack of data.

By reducing the noise level in the signal, while minimally affecting the evoked potential signal using the GGMRF technique and considerably increasing the signal-to-noise ratio (Fig 9), this framework increases the detection accuracy of the earliest muscle response in the EMG signal and the corresponding stimulation voltage (intensity) as the muscle activation threshold. In other words, this algorithm detects the exact intensity threshold that is needed for the muscle to get activated (for a given electrode configuration). Determining this threshold value has two main advantages for the experimentalist: 1) To find the configuration (or combination of several configurations) that offers the lowest intensity threshold for activating all the muscles; and 2) To set the intensity at the pre-threshold value during the performance of specific voluntary tasks for the selected optimum configuration. Therefore, the automatic process of linking scES parameters to the muscle recruitment order that is presented in this study improves the speed and precision of the operator’s decision for selecting both the optimum configuration and the corresponding pre-threshold intensity.

Finally, our framework has the flexibility to be applied to any other experimental protocols or signals, by simply updating certain parameters (e.g. intensity or frequency) and attributes (e.g. peak-to-peak amplitude or integrated EMG) of the signals. Spinal cord epidural stimulation research has current applications in small and large animal models as well as human models. In all models, understanding the effect of scES on the spinal cord networks following injury is a critical component that will lead to more successful selection of stimulation parameters targeted for functional improvements. As we have shown in our previous works (3–6) stimulation configurations are different across individuals, species and tasks, emphasizing the need to map motor evoked responses relative to stimulation site for each research participant. Transcutaneous stimulation has also been used as a technique to access the capabilities of the spinal cord networks following injury. Thus, the methodology presented in this paper can also be used to visualize the motor evoked responses relative to stimulation site and stimulation voltage across multiple muscles. Applications in which understanding detailed responses of multiple muscles to a stimulus with varying intensity and location could benefit from the analysis technique explained in this paper.

## Conclusions

In this study, we presented a novel framework consisting of five algorithms for activation detection and visualization of EMG signals recorded from multiple leg muscles of spinal cord injured subjects with an epidural stimulator implanted in lumbosacral region of their spine. Using this framework, raw EMG signals are successfully converted into two/three dimensional images and de-noised using GGMRF image smoothing technique. Additionally, the occurrence of scES induced muscle activation is automatically detected along with the ability to extract key features in the EMG signal and generate the visual output for user interpretation.

Each of these five novel algorithms has several advantages over conventional methodologies, which make them indispensable for the data analysis application. For instance, the first algorithm converts the signal into an image, which enables clear illustration of the latencies for all activations as well as the overall onset of the scES induced motor responses. By converting the raw signal into image, this algorithm also prepares the data for the next step that is GGMRF image smoothing method to get a de-noised signal without affecting the shape and latency of muscle activations. As it is shown in the results section adding the GGMRF method to the framework is also noticeably advantageous for the performance of the activation detection step. In the activation detection algorithm, we developed a statistically optimal decision method by applying MLE together with comparing the probability density functions of the muscle activations to the background noise utilizing LLR and calculating the dynamic activation threshold. In comparing the automatic method for activation threshold detection vs. manual detection (ground truth) on 700 EMG signals, the new automated approach developed here demonstrated average accuracy of 98.28% based on the errors of combined false positive and false negative data.

Finally, the combination of the modified AGLR method and GGMRF has proven to have minimum sensitivity to the changes in the signal to noise ratio compared to the other well-known EMG onset detection methods i.e. AGLR method without smoothing and TKEO method using both simulated EMG signal and real EMG signals. Comparing the three methods on the recorded EMG signals indicates the robustness in the accuracy of the presented activation method in the situation where no information about the noise level in the signal is known. In addition, the feature extraction and visualization steps of the framework help us to make accurate and quick connections between the desired EMG features and the scES parameters like intensity voltage, configuration and frequency. In conclusion, this study clearly demonstrates the advantages of implementing a set of algorithms for improving the accuracy and speed in complex EMG data analysis.

## Supporting information

S1 Fig Simulated EMG signal. (A) Process of generating the simulated signal; (B) examples of the simulated signals after adding the white Gaussian noise with SNR(dB) = 5,0 and -10 (top figures) and the output of the GGMRF algorithm to show to what extend this method reduces the noise level in the noisy signals.
(TIF)

S1 Table Comparison of the performance measurements for the automated activation detection method based on the manual detection as the gold standard. These values are calculated based on signals recorded from 14 leg muscles for all five participants during intensity ramp-up experiments. The five-number-summery values presented here are maximum, median, upper and lower quartile, and minimum values of all the measurements.
(DOCX)

S2 Table comparing the automated activation detection method with the manual ground truth as separated by subjects. (DOCX)

S3 Table comparing the automated activation detection method with the manual ground truth as separated by muscles. (DOCX)

S1 Appendix Interpolation process. (DOCX)

S2 Appendix Pseudocodes of all algorithms in the framework. (DOCX)
