# Supplementary figures and images for: A novel approach for automatic visualization and activation detection of evoked potentials induced by epidural spinal cord stimulation in individuals with spinal cord injury
Source: PLoS One. 2017 Oct 11;12(10):e0185582. doi: 10.1371/journal.pone.0185582 (PMC5636093; doi:10.1371/journal.pone.0185582)

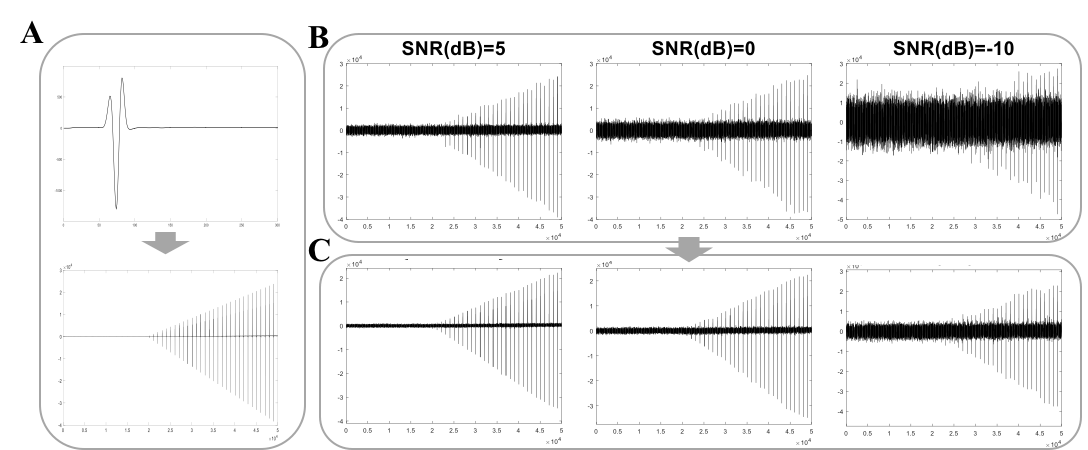

Supplement: S1 Fig — (A) Process of generating the simulated signal; (B) examples of the simulated signals after adding the white Gaussian noise with SNR(dB) = 5,0 and -10 (top figures) and the output of the GGMRF algorithm to show to what extend this method reduces the noise level in the noisy signals. (TIF) [file pone.0185582.s001.tif]
