# Supplementary material for: A novel approach for automatic visualization and activation detection of evoked potentials induced by epidural spinal cord stimulation in individuals with spinal cord injury
Source: PLoS One. 2017 Oct 11;12(10):e0185582. doi: 10.1371/journal.pone.0185582 (PMC5636093; doi:10.1371/journal.pone.0185582)
Supplement: S1 Table — These values are calculated based on signals recorded from 14 leg muscles for all five participants during intensity ramp-up experiments. The five-number-summery values presented here are maximum, median, upper and lower quartile, and minimum values of all the measurements. (DOCX) [file pone.0185582.s002.docx]

**S1 Table.** Comparison of the performance measurements for the automated activation detection method based on the manual detection as the gold standard. The five-number-summary of all the calculated values are presented.

|  | **Total Accuracy %**  **(TP+TN)/(TP+FP+TN+FN)** | **Total Sensitivity %**  **TP/(TP + FN)** | **Total Specificity %**  **TN/(TN+FP)** | **Total Dice Similarity %**  **2TP/(2TP+FN+FP)** |
| --- | --- | --- | --- | --- |
| **Maximum** | 100.00 | 99.99 | 99.99 | 99.99 |
| **Upper quartile** | 100.00 | 99.99 | 99.99 | 99.99 |
| **Median** | 100.00 | 99.99 | 99.99 | 99.99 |
| **Lower quartile** | 97.72 | 99.99 | 91.66 | 98.46 |
| **Minimum** | 94.33 | 99.99 | 79.59 | 96.29 |

These values are calculated based on signals recorded from 14 leg muscles for all five participants during intensity ramp-up experiments. This table shows the five-number-summary of all the comparison measurements. These five values are maximum, median, upper and lower quartile, and minimum values of all the measurements. The five-number-summary representation is usually used in the literature to present data that do not follow a certain parametric distribution. It is notable that in this method the values that are three times less than the lower quartile are considered as outliers and are not used for calculating the minimum. The five-number-summary can also be presented graphically using boxplot.
