# Supplementary material for: A novel approach for automatic visualization and activation detection of evoked potentials induced by epidural spinal cord stimulation in individuals with spinal cord injury
Source: PLoS One. 2017 Oct 11;12(10):e0185582. doi: 10.1371/journal.pone.0185582 (PMC5636093; doi:10.1371/journal.pone.0185582)
Supplement: S2 Table — (DOCX) [file pone.0185582.s003.docx]

**S2 Table.** Five-number-summary of the performance measurements for comparing the automated activation detection method with the manual ground truth as a function of subjects

| **Subject #** |  | **1** | **2** | **3** | **4** | **5** |
| --- | --- | --- | --- | --- | --- | --- |
| **Sensitivity %** | **Max** | 99.9998 | 99.9998 | 99.9998 | 99.9999 | 99.9999 |
|  | **Upper** | 99.9998 | 99.9997 | 99.9997 | 99.9998 | 99.9997 |
|  | **Med** | 99.9997 | 99.9997 | 99.9997 | 99.9998 | 99.9997 |
|  | **Lower** | 99.9997 | 99.9996 | 99.9995 | 99.9996 | 99.9996 |
|  | **Min** | 99.9995 | 99.9995 | 99.9993 | 99.9996 | 99.9994 |
| **Specificity %** | **Max** | 99.9998 | 99.9996 | 99.9999 | 99.9994 | 99.9995 |
|  | **Upper** | 99.9982 | 99.999 | 99.9992 | 99.999 | 99.9989 |
|  | **Med** | 99.9967 | 99.9986 | 99.9982 | 99.9975 | 99.9967 |
|  | **Lower** | 88.8879 | 99.9975 | 93.3327 | 99.99 | 85.1639 |
|  | **Min** | 72.7266 | 99.9967 | 85.4165 | 99.99 | 66.6644 |
| **Similarity %** | **Max** | 99.9999 | 99.9999 | 99.9999 | 99.9999 | 99.9999 |
|  | **Upper** | 99.9999 | 99.9999 | 99.9999 | 99.9999 | 99.9999 |
|  | **Med** | 99.9998 | 99.9998 | 99.9997 | 99.9998 | 98.999 |
|  | **Lower** | 98.6300 | 98.5914 | 98.1648 | 99.1869 | 97.8248 |
|  | **Min** | 96.6665 | 96.5516 | 95.9998 | 98.039 | 94.7367 |
| **Accuracy %** | **Max** | 100.0000 | 100.0000 | 100.0000 | 100.0000 | 100.0000 |
|  | **Upper** | 100.0000 | 100.0000 | 100.0000 | 100.0000 | 100.0000 |
|  | **Med** | 100.0000 | 100.0000 | 98.9583 | 100.0000 | 98.2143 |
|  | **Lower** | 97.7778 | 97.9167 | 97.2973 | 98.6842 | 97.1429 |
|  | **Min** | 95.0000 | 95.4545 | 94.5946 | 96.875 | 93.0233 |
