# Supplementary material for: A novel approach for automatic visualization and activation detection of evoked potentials induced by epidural spinal cord stimulation in individuals with spinal cord injury
Source: PLoS One. 2017 Oct 11;12(10):e0185582. doi: 10.1371/journal.pone.0185582 (PMC5636093; doi:10.1371/journal.pone.0185582)
Supplement: S3 Table — (DOCX) [file pone.0185582.s004.docx]

**S3 Table.** Five-number-summary of the performance measurements for comparing the automated activation detection method with the manual ground truth as a function of muscles

|  |  | **L SOL** | **L MG** | **L TA** | **L MH** | **L VL** | **L RF** | **L GL** | **R SOL** | **R MG** | **R TA** | **R MH** | **R VL** | **R RF** | **R GL** |
| --- | --- | --- | --- | --- | --- | --- | --- | --- | --- | --- | --- | --- | --- | --- | --- |
| **Sensitivity %**  **TP/(TP + FN)** | **Max** | 99.9999 | 99.9999 | 99.9999 | 99.9999 | 99.9999 | 99.9999 | 99.9998 | 99.9999 | 99.9999 | 99.9999 | 99.9999 | 99.9998 | 99.9998 | 99.9998 |
|  | **Upper** | 99.9998 | 99.9998 | 99.9998 | 99.9998 | 99.9998 | 99.9998 | 99.9998 | 99.9997 | 99.9998 | 99.9997 | 99.9998 | 99.9998 | 99.9998 | 99.9998 |
|  | **Med** | 99.9997 | 99.9997 | 99.9997 | 99.9997 | 99.9997 | 99.9997 | 99.9997 | 99.9996 | 99.9997 | 99.9997 | 99.9997 | 99.9997 | 99.9997 | 99.9997 |
|  | **Lower** | 99.9996 | 99.9996 | 99.9996 | 99.9997 | 99.9997 | 99.9996 | 99.9996 | 99.9995 | 99.9995 | 99.9995 | 99.9996 | 99.9996 | 99.9996 | 99.9996 |
|  | **Min** | 99.9995 | 99.9995 | 99.9995 | 99.9996 | 99.9995 | 99.9995 | 99.9996 | 99.9995 | 99.9993 | 99.9992 | 99.9996 | 99.9995 | 99.9995 | 99.9995 |
| **Specificity%**  **TN/(TN + FP)** | **Max** | 99.9998 | 99.9996 | 99.9997 | 99.9995 | 99.9995 | 99.9997 | 99.9992 | 99.9996 | 99.9999 | 99.9998 | 99.9998 | 99.9995 | 99.9995 | 99.9993 |
|  | **Upper** | 99.999 | 99.9988 | 99.9989 | 99.9989 | 99.9989 | 99.9991 | 99.9988 | 99.9993 | 99.9992 | 99.9992 | 99.9989 | 99.999 | 99.9991 | 99.9986 |
|  | **Med** | 99.998 | 99.9975 | 99.9975 | 99.998 | 99.9958 | 99.9983 | 94.0345 | 99.9988 | 99.9978 | 99.9958 | 99.9975 | 99.9978 | 99.9973 | 96.4283 |
|  | **Lower** | 99.995 | 99.99 | 94.3394 | 95.3486 | 89.9991 | 91.304 | 76.9225 | 99.995 | 94.9995 | 88.8879 | 89.9991 | 94.4439 | 88.8879 | 85.7131 |
|  | **Min** | 99.99 | 99.99 | 91.304 | 88.8879 | 79.9984 | 83.3319 | 49.9996 | 99.99 | 88.8879 | 76.4701 | 76.1901 | 88.8879 | 77.7773 | 66.6656 |
| **Dice Similarity%**  **2TP/(2TP+FP+FN)** | **Max** | 99.9999 | 99.9999 | 99.9999 | 99.9999 | 99.9999 | 99.9999 | 99.9999 | 99.9999 | 99.9999 | 99.9999 | 99.9999 | 99.9999 | 99.9999 | 99.9999 |
|  | **Upper** | 99.9999 | 99.9999 | 99.9999 | 99.9999 | 99.9999 | 99.9999 | 99.9998 | 99.9999 | 99.9999 | 99.9999 | 99.9999 | 99.9999 | 99.9999 | 99.9999 |
|  | **Med** | 99.9998 | 99.9998 | 99.9998 | 99.9998 | 99.9998 | 99.9998 | 98.8228 | 99.9998 | 99.9998 | 99.6451 | 99.9998 | 99.9998 | 99.9998 | 99.0461 |
|  | **Lower** | 99.9998 | 98.5914 | 98.1816 | 98.5914 | 98.5506 | 98.63 | 97.6189 | 98.4614 | 98.3605 | 98.1816 | 98.4848 | 98.8505 | 98.3049 | 97.8721 |
|  | **Min** | 99.9998 | 96.8748 | 96.2961 | 96.7032 | 97.1427 | 96.8748 | 95.2379 | 96.2961 | 97.297 | 95.652 | 97.959 | 97.7776 | 96.2961 | 95.4544 |
| **Accuracy %**  **(TP+TN)/ (FP+FN+TP+TN)** | **Max** | 100.00 | 100.00 | 100.00 | 100.00 | 100.00 | 100.00 | 100.00 | 100.00 | 100.00 | 100.00 | 100.00 | 100.00 | 100.00 | 100.00 |
|  | **Upper** | 100.00 | 100.00 | 100.00 | 100.00 | 100.00 | 100.00 | 100.00 | 100.00 | 100.00 | 100.00 | 100.00 | 100.00 | 100.00 | 100.00 |
|  | **Med** | 100.00 | 100.00 | 100.00 | 100.00 | 100.00 | 100.00 | 98.0769 | 100.00 | 100.00 | 99.3421 | 100.00 | 100.00 | 100.00 | 98.4835 |
|  | **Lower** | 100.00 | 97.6744 | 97.1429 | 97.9167 | 97.7273 | 97.9167 | 96.0526 | 97.7273 | 97.7273 | 97.561 | 97.9167 | 98.2143 | 97.6744 | 96.1538 |
|  | **Min** | 100.00 | 94.2857 | 93.0233 | 95.0000 | 95.8333 | 95.0000 | 93.1818 | 95.8333 | 94.5946 | 94.2857 | 97.1429 | 95.8333 | 94.3396 | 91.4286 |
