# Supplementary material for: A novel approach for automatic visualization and activation detection of evoked potentials induced by epidural spinal cord stimulation in individuals with spinal cord injury
Source: PLoS One. 2017 Oct 11;12(10):e0185582. doi: 10.1371/journal.pone.0185582 (PMC5636093; doi:10.1371/journal.pone.0185582)
Supplement: S1 Appendix — (DOCX) [file pone.0185582.s005.docx]

A Novel Approach for Automatic Visualization and Activation Detection of Evoked Potentials Induced by Epidural Spinal Cord Stimulation in Individuals with Spinal Cord Injury

Samineh Mesbah, Claudia A. Angeli, Robert S. Keynton, Ayman El-baz^*^, Susan J. Harkema

*** Correspondence:** Dr. Ayman El-baz: ayman.elbaz@louisville.edu

# S1 Appendix. Interpolation Process

In most of the executed voltage ramp-up experiments, there are always some stimulation intensities that are overlooked, especially after the muscle groups start to activate during the voltage ramp-up phase of the experiments. For example, the stimulation intensity starts at a low voltage value and ramps-up at 0.1 V increments. If the muscle groups are activated, the ramp-up voltage increment is increased from 0.1 to 0.5 V until a maximum stimulation potential of 10 V, or the highest intensity value in which the patient feels comfortable, is achieved. As a result, there are instances where discontinuities between data points corresponding to the overlooked stimulation voltages can occur. Therefore, in order to maintain the continuity and resolution of the Colormap images in the visualization step, the missing values are interpolated based on the observed values. This task is performed using a cubic spline smoothing method. Assume that there is a given set of coordinates (*x_0_, y_0_*), (*x_1_, y_1_*), ..., (*x_n_, y_n_*) where the values *x_i_* are stimulation voltages in ascending order and *y_i_* are any quantitative feature of the evoked potentials. The cubic spline smoothing method links the “gap” between adjacent points (*x_i_, y_i_*), (*x_i+1_, y_i+1_*) using cubic functions (*P_i_*: *i = 0, ... ,n−1*) in order to piece together the curve using functions that have continuous first- and second-order derivatives. In other words, this technique tends to preserve the values of the observed data while interpolating the missing values using a piecewise curve function that must satisfy the end points matching conditions [[1](#Pol99)]. The cubic spline smoothing method allows selection of a smoothing parameter $p$, which determines the level of closeness of the function estimate to the data and takes any values between 0 and 1. The choice of $p=0$ makes *P_i_* least squares straight line fit to the data, whereas with $p=1$ the function estimate is the closest to the data and is called the natural cubic spline interpolant. In this study, $p=1$ was selected in order to preserve a smooth, yet accurately interpolated curve (Algorithm V in S2 Appendix).
