# Supplementary material for: A novel approach for automatic visualization and activation detection of evoked potentials induced by epidural spinal cord stimulation in individuals with spinal cord injury
Source: PLoS One. 2017 Oct 11;12(10):e0185582. doi: 10.1371/journal.pone.0185582 (PMC5636093; doi:10.1371/journal.pone.0185582)
Supplement: S2 Appendix — (DOCX) [file pone.0185582.s006.docx]

A Novel Approach for Automatic Visualization and Activation Detection of Evoked Potentials Induced by Epidural Spinal Cord Stimulation in Individuals with Spinal Cord Injury

Samineh Mesbah, Claudia A. Angeli, Robert S. Keynton, Ayman El-baz^*^, Susan J. Harkema

*** Correspondence:** Dr. Ayman El-baz: ayman.elbaz@louisville.edu

**S2 Appendix. Pseudocodes of All Algorithms in the Framework**

| **Algorithm I:** 2-D representation of the raw EMG signals |
| --- |
| - Find the time intervals between each two consecutive stimulation pulsations using the onset timing of each stimulation - Use the time intervals to segment the EMG signals into corresponding pieces - Repeat for all the muscles - Save the samples of EMG segments into a $m\times n\times l$ matrix where *m* is the number of stimulation pulsation, *n* is the maximum time interval between consecutive stimulations, and *l* is the number of muscles. - Use *imagesc* function to visualize each $m\times n$ matrix as Colormap image - Repeat for all muscles |

| **Algorithm II:** Noise reduction |
| --- |
| - Initialize the GGMRF parameters $\boldsymbol{\sigma}$, $\boldsymbol{\lambda}$, $\boldsymbol{p}$, $\boldsymbol{q}$, and $\boldsymbol{b}_{\boldsymbol{s,r}}$ for Eq. 2.1 - Determine the window size for the Eq. 2.1 - Design an all ones $m\times n$ mask matrix and set its four borders rows and columns to zero - Use $m\times n$ segmentation matrix, $m\times n$ mask matrix, parameters and window size to substitute in Eq. 2.1 and calculate the estimated value for the pixel - Repeat for all pixels - Repeat for all muscles |

| **Algorithm III:** Activation detection using SOD method |
| --- |
| - Select the background noise as the first non-zero segment of the de-noised EMG signal - Use *mle* function to estimate the Gaussian distribution parameters $\sigma_{0}$ and $\mu_{0}$ - For each segment of the de-noised signal use *mle* function to estimate the Gaussian distribution parameters $\sigma_{i}$ and $\mu_{i}$ - Calculate $S_{i}$ using Eq. 2.4 for all segments *i* of the de-noised signal - Select the baseline as the first event of the de-noised EMG signal - Calculate $S_{max}$ and $\sigma_{S_{i}}$ for the selected baseline to find the activation threshold *h* using Eq. 2.5 - Compare each $S_{i}$ with *h*: **If** $S_{i}>h$, detect the evoked potential in the $i^{th}$ segment and represent it with 1, **else** there is no evoked potential and represent it with 0 - **If** 50% of the segments inside one *event* is active call the whole *event* active, **else** call it inactive - Repeat for all *events* - Find the corresponding stimulation intensity to the first active *event* and assign it as the voltage threshold - Repeat for all muscles |

| **Algorithm IV:** Feature extraction |
| --- |
| **Peak-to-peak and min-max interval:**   - Divide each segment into 8 pieces and find the maximum $A_{max}$ and minimum $A_{min}$ amplitude values inside the first piece and their corresponding timings $T_{max}$ and $T_{min}$. - Calculate the peak-to-peak value ($A_{pp}=A_{max}-A_{min}$) and min-max interval ($T_{mm}=\left\vert T_{max}-T_{min} \right\vert$) for each segment $i$ - Repeat for all segments - Take the average over all $A_{pp}$ and $T_{mm}$ values inside one *event* - Repeat for all *events* - Repeat for all muscles   **Activation latency:**   - Up-sampling the signal to 100,000 samples per second using function interp1. - Divide each segment, which are detected as active by Algorithm III, into 20 pieces and take the first five pieces as baseline - Apply the same method in Algorithm III to detect the onset timing of the evoked potential in each segment $L_{i}$ - Repeat for all segments - Take the average over all $L_{i}$ inside one *event* $L$ - Repeat for all *events* - Repeat for all muscles   **Integrated EMG:**   - Rectify each segment of the EMG signal - Take the integral of the rectified signal $I_{EMG}$ - Repeat for all segments - Take the average over all $I_{EMG}$ values inside one *event* - Repeat for all *events* - Repeat for all muscles |

| **Algorithm V:** Visualization |
| --- |
| - Use *csaps* function (cubic smoothing spline) to interpolate the missing values for $A_{pp}$, $T_{mm}, L$ and $I_{EMG}$ - Assign each feature values to the corresponding stimulation intensity voltage $A_{pp}$ - Repeat for all *events* - Repeat for all the muscles - Use *imagesc* function to visualize each matrix as Colormap image |

| **Algorithm VI:** Activation detection using TKEO method |
| --- |
| - Segment the signal based on stimulation timings (similar to Algorithm I) - Calculate the TEKO value for each sample $x_{ijk}$ inside the segment ${TKEO}_{ijk}= {x_{ijk}}^{2}-{(x}_{i-1jk}{.x}_{i+1jk})$ - Repeat for all segments $j$ - Repeat for all muscles $k$ - Take the first *event* after TKEO operation as baseline - Calculate the baseline maximum and standard deviation and find the activation threshold $h={{TKEO}_{base}}_{max}+{TKEO}_{sd}$ - Calculate the maximum value for the segment ${TKEO}_{max}$ - **If** ${TKEO}_{max}>h$, detect the evoked potential in the current segment and represent it with 1, **else** there is no evoked potential and represent it with 0 - Repeat for all segments - **If** 50% of the segments inside one *event* is active call the whole *event* active, **else** call it inactive - Repeat for all *events* - Find the corresponding stimulation intensity to the first active *event* and assign it to be the activation intensity threshold - Repeat for all the muscles |
